# A six-week balance training intervention with older inmates improves static balance in a German prison

**DOI:** 10.1016/j.jarlif.2026.100065

**Published:** 2026-02-16

**Authors:** Milan Dransmann, Martin Koddebusch, Bernd Gröben, Pamela Wicker

**Affiliations:** Department of Sports Science, Bielefeld University, Universitaetsstr. 25, Bielefeld 33615, Germany

**Keywords:** Dynamic balance, Unstable surfaces, Well-being, Physical activity enjoyment, Control group

## Abstract

**Introduction:**

Given the accelerated aging processes and elevated fall risk associated with functional decline in correctional settings, developing effective exercise programs for older inmates is increasingly important.

**Methods:**

This study examined the potential effects of a six‑week balance‑training program on static and dynamic balance performance, well‑being, and enjoyment of physical activity among older inmates in an open German prison. Ten male inmates (mean age = 62.1 ± 4.4 years) participated, with five assigned to an intervention group and five to a control group. The intervention comprised three 60‑minute sessions per week. based on a standardized balance‑training protocol for older adults. A repeated‑measures design assessed pre‑ and post‑intervention changes in anthropometric data, static balance, static balance on unstable surfaces, dynamic balance, well‑being, and physical activity enjoyment.

**Results:**

The training group showed significant improvements in static balance and static balance on unstable surfaces, corresponding to functional gains of approximately 26% and 33%, respectively. Dynamic balance, well‑being, and enjoyment of physical activity did not change significantly.

**Discussion:**

The observed improvements in static balance suggest that even limited interventions may yield meaningful physical benefits in older inmates. However, the absence of effects on dynamic balance and psychosocial outcomes may reflect the short intervention duration, small sample size, and ongoing stressors inherent to incarceration. Future studies should extend program length and include follow‑up assessments to evaluate long‑term benefits.

**Conclusion:**

A short, resource‑efficient balance‑training program was associated with improved balance performance in older inmates and may help preserve mobility, autonomy, and quality of life in aging prison populations.

## 1 Introduction

In Germany, the growing number of older adults is expected to increase the need for health care and support services [1]. Although this issue has been widely discussed, aging in correctional institutions has received comparatively little research attention. Although criminal behavior is most common among young adults, the proportion of older inmates has increased significantly over the past decades. In Germany, the share of prisoners aged 60 years and older has more than tripled since the 1990s, indicating that aging has become an important structural feature of the prison population [2].

Imprisonment represents a severe psychological and physical burden for all individuals, but its impact is particularly pronounced in older age. Younger inmates can often compensate for losses experienced during imprisonment, whereas older inmates have fewer opportunities to adapt to the challenges of incarceration. Their needs therefore differ considerably from those of younger inmates and require age‑appropriate infrastructure, health care, and recreational programs [3]. In correctional contexts, individuals are often considered older from about 50 years onward, because imprisonment tends to accelerate physical and psychological aging processes [4]. Mental disorders and infectious diseases are more common in prisoners than in the general population, and higher suicide and mortality rates have been reported [5].

Despite the growing proportion of older inmates, German correctional facilities still lack systematic strategies to address aging‑related needs. Health promotion programs remain scarce, and palliative care is largely absent [6]. Sport and exercise in prisons can, however, play an important socioeducational role by promoting social integration, and group cohesion [7], particularly when guided by trained coaches [8].

Physical inactivity and the restrictive conditions of incarceration accelerate aging, leading to loss of strength, balance, and independence and thereby increasing vulnerability to falls, a major cause of injury and declining autonomy among older adults [9]. Incarcerated adults experience accelerated aging, a process in which exposure to incarceration speeds up biological aging, further increasing their vulnerability to health decline [10]. Studies suggest that inmates age physiologically 10–15 years faster than individuals in the general population [11], making targeted exercise interventions particularly relevant.

Evidence from community‑dwelling older adults shows that balance training can improve static and dynamic balance performance [12] and enhance psychological well‑being [13]. Moreover, such interventions can increase quality of life [14] and enjoyment of physical activity [15]. Previous research in prisons has investigated balance only as a secondary outcome within general fitness [16] or strength and endurance programs [17]. No study to date has explicitly focused on balance training in prison populations, particularly among older inmates.

Prison environments pose specific challenges for implementing and sustaining physical activity interventions due to restricted facilities, regimented daily routines, and psychosocial stressors [18]. Structural barriers and limited opportunities for health promotion can further exacerbate functional decline and accelerate aging processes [19]. Investigating balance training in this context is therefore essential to determine whether established community approaches can be effectively transferred and adapted to correctional settings.

The present study aimed to examine whether a six‑week balance‑training intervention could improve static and dynamic balance performance, well‑being, and enjoyment of physical activity among older inmates in an open German prison. It was hypothesized that participation in the training program would enhance balance performance and be positively associated with psychological outcomes.

## 2 Methods

### 2.1 Participants

The study was conducted in cooperation with an open prison in Germany as part of a project promoting physical activity among older inmates. Participants were recruited through information sessions held by the university. Participation was voluntary, and all inmates aged 55 years and older were invited to take part if their health status allowed engagement in physical exercise, as determined by the prison administration. Because only men are incarcerated in this facility, all participants in the study were male. Of the inmates who expressed interest (n = 14), eight chose to participate in the intervention group and six in the control group. All participants were serving their sentences in an open facility, allowing them to leave the prison for work. Depending on individual behavior and detention level, temporary leaves of several hours or days were also possible. Five of the eight participants in the intervention group completed the six‑week training, whereas three dropped out due to exceeding the maximum allowed absenteeism. One inmate from the control group was released early. Consequently, ten inmates – five in the control group (age 61.6 ± 4.28 years) and five in the intervention group (62.6 ± 4.72 years) – were included in the empirical analysis. Before the start of the program, all participants were informed about the study design and content, data‑handling procedures, and their right to withdraw before providing written informed consent. Inclusion criteria required attendance of at least 80% of training sessions and a health status sufficient to engage in physical activity. Neither physical examination nor physician clearance was conducted. All procedures complied with the Declaration of Helsinki, and the study was approved by the Ethics Committee of Bielefeld University (approval number 2022‑193).

### 2.2 Design and procedure

**Fig. 1:**
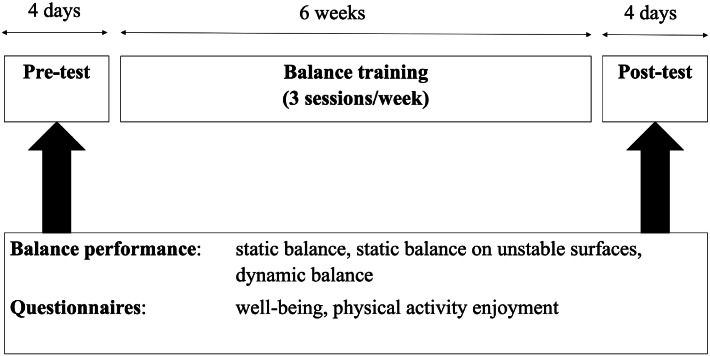
The timeline and outcome variables of this study.

The investigation followed a pre‑post design comprising a six‑week balance‑training intervention. Training sessions were conducted three times per week. Fig. 1 provides an overview of the study design and the outcome variables. Pre‑ and post‑tests assessed each participant’s static balance, static balance on unstable surfaces, and dynamic balance, as well as well‑being and enjoyment of physical activity using standardized questionnaires. Baseline testing (pre‑test) was performed 96 hours before the first training session, and post‑test 96 hours after the final session. Both assessments took place at the same time of day to ensure comparability. Testing was supervised by the scientific project manager and three trained test assistants who followed standardized instructions. All measurements were conducted indoors.

### 2.3 Intervention

The balance‑training program for the intervention group was based on the established protocol by Granacher et al. [20] and was adapted to the specific conditions and fitness level of the participating inmates according to the principles of coordination and balance training in older adults [21]. The intervention lasted six weeks and consisted of three sessions per week, resulting in a total of 18 training sessions of approximately 60 minutes each. Each training session included a ten‑minute warm‑up with brisk walking and joint mobilization, a forty‑minute balance‑training phase, and a ten‑minute cool‑down with light walking. The training was organized in a circuit format comprising four stations, each representing one piece of balance equipment. Participants completed eight sets per station, performing the first four sets bipedally and the remaining four unipedally, alternating legs after each set. The difficulty of the exercises increased progressively by narrowing the base of support, closing the eyes, or using unstable surfaces.

### 2.4 Measurements

Body height was measured at baseline with a stadiometer, and body mass was measured before and after the intervention using a scale. Balance performance was assessed with the standardized GGT‑Reha test battery [22], which provides a multidimensional evaluation of postural control under static and dynamic conditions. The instrument was originally developed for use in rehabilitation settings with participants aged 20 to 76 years, most of whom were undergoing neurological rehabilitation. The GGT‑Reha requires minimal equipment, can be administered efficiently, and allows the simultaneous testing of several participants – an important advantage in the prison environment. The test comprises three progressively demanding blocks with 18 tasks that together capture static balance on stable and unstable surfaces and dynamic balance. Each task is rated on a four‑point scale (0–3 points) according to execution quality, with higher scores indicating better balance performance. All tests were administered following the standardized protocol of the GGT‑Reha. According to Theisen and Wydra [22], the test battery demonstrates excellent inter‑tester reliability (r = 0.98) and test‑retest reliability (r = 0.94). Reported validity coefficients compared with posturographic methods range from r = 0.55 to r = 0.47, indicating moderate criterion validity for the total score. The authors attribute this moderate validity to the multidimensional nature of the GGT‑Reha, which assesses different components of balance ability rather than a construct. In the present study, all performances were independently evaluated by three trained test assistants to ensure high inter‑rater objectivity. Inter‑rater reliability across the three raters was r = 0.92, indicating excellent rating consistency [23]. In addition, the Single-Leg Stance (SLS) test (also referred to as the Unipedal Stance Test, UPST) [24] was assessed to capture static balance performance.

### 2.5 Questionnaires

Psychological outcomes were assessed using two standardized self‑report instruments. General well‑being was measured with the WHO‑5 Well‑Being Index [25], which includes five positively phrased items rated on a six‑point scale from 0 to 5; higher total scores indicate better well‑being. The WHO‑5 has demonstrated good reliability and validity across various populations, including inmates [26]. Enjoyment of physical activity was measured with the Physical Activity Enjoyment Scale (PACES) [27], which consists of 16 items rated on a five‑point Likert scale from 1 to 5; higher scores indicate greater enjoyment. The PACES has demonstrated good reliability and validity among older adults and is considered an appropriate measure of exercise enjoyment in this population [28].

### 2.6 Statistical analyses

The empirical analyses were carried out in SPSS 30 and consisted of two main steps. First, descriptive statistics of all variables were obtained, including mean and standard deviation. Exploratory data analysis was performed to assess distributions, identify outliers, and evaluate potential ceiling or floor effects. Boxplots and distribution checks were used to support assumptions for parametric analyses. Second, analyses of variance (ANOVA) were conducted to test the hypotheses outlined in the introduction. All variables were tested for normal distribution using the Shapiro–Wilk test, which indicated that all were normally distributed (p > 0.05). To analyze potential changes for each outcome variable (see Fig. 1) from pre‑test to post‑test, a two‑group (intervention, control) × two‑time (pre, post) repeated‑measures analysis of variance (ANOVA) was performed. Post‑hoc analyses (independent group and pairwise comparisons) were conducted using the Bonferroni correction. An alpha level of 0.05 was used for all statistical tests. In addition to statistical significance, effect sizes were reported using partial eta‑squared (ηp²).

## 3 Results

All five participants in the intervention group completed an average of 17.2 ± 1.2 training sessions without any adverse events. Baseline characteristics and outcome measures were broadly comparable between the intervention and control groups, and no missing data occurred.

### 3.1 Anthropometric data

Table 1 summarizes the anthropometric results. No significant changes in body mass were observed from pre‑ to post‑test in either the intervention or the control group, and no differences were found between groups for body height or body mass.

**Table 1:** Anthropometric parameters (M ± SD) before and after the intervention (*n* = 10).

Parameter	Group	Pre-test	Post-test	*F*	*p*	*ηp2*
Body mass [kg]	Intervention	84.84 ± 17.56	84.52 ± 16.52	0.171	0.701	0.041
	Control	84.66 ± 10.02	85.06 ± 9.12	0.389	0.566	0.089
Body height [cm]	Intervention	174.60 ± 6.11	-	-	-	-
	Control	176.60 ± 5.73	-	-	-	-

### 3.2 Balance performance

**Fig. 2:**
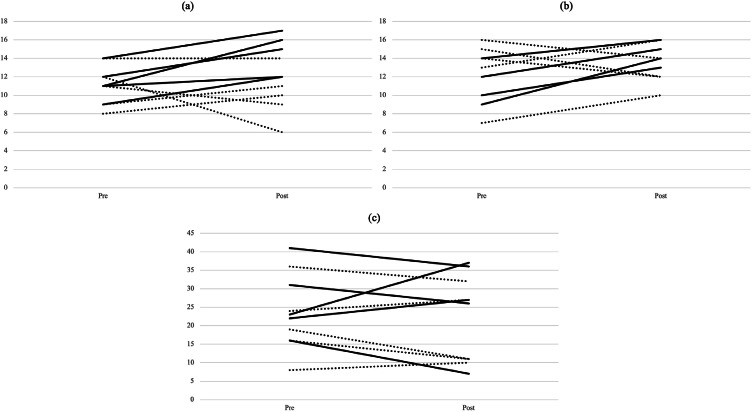
Pre‑ and post‑intervention balance performance by group. Panels show (a) static balance, (b) static balance on unstable surfaces, and (c) dynamic balance. Each line represents one participant; line style distinguishes intervention (solid line) and control group (dashed line).

As illustrated in Fig. 2, the line plots depict participant trajectories, indicating considerable variability within both groups but also a consistent overall improvement in balance performance following the intervention. This visualization allows for a more nuanced understanding of individual response patterns and group-level trends beyond mean differences. Table 2 presents the results of the balance performance tests. For static balance, the repeated‑measures analysis of variance showed no significant main effect of time (p = .213) but a significant time‑by‑group interaction (F(1, 8) = 5.47, p = .048, ηp² = .41). Post‑hoc comparisons indicated a significant improvement in the intervention group from pre‑ to post‑test (p = .031, ηp² = .46), whereas no change was observed in the control group (p = .51, ηp² = .06). For static balance on unstable surfaces, both the main effect of time (p = .047) and the time‑by‑group interaction were significant (F(1, 8) = 6.88, p = .031, ηp² = .46). Participants in the training group improved significantly from pre‑ to post‑test (p = .008, ηp² = .61), whereas the control group showed no significant change (p = .85, ηp² = .01). Dynamic balance results, also shown in Table 2, revealed no significant differences across time or between groups (F(1, 8) = 0.26, p = .62, ηp² = .03), indicating that performance remained stable in both groups.

**Table 2:** Balance performance (M ± SD) before and after the intervention (*n* = 10).

Parameter	Group	Pre-test	Post-test	*F*	*p*	*ηp2*
Static balance	Intervention	11.40 ± 1.82	14.40 ± 2.30	22.500	0.009	0.849
	Control	10.80 ± 2.39	10.00 ± 2.92	0.286	0.621	0.067
Static balance on unstable surfaces	Intervention	10.80 ± 2.17	14.40 ± 1.14	36.000	0.004	0.900
	Control	13.00 ± 3.54	12.80 ± 2.28	0.023	0.887	0.006
Dynamic balance	Intervention	26.60 ± 9.66	26.60 ± 12.05	0.000	1.000	0.000
	Control	20.60 ± 10.38	18.20 ± 10.47	1.291	0.319	0.244

However, the results of the SLS test were not included in the inferential analyses because the data deviated significantly from normality (Shapiro–Wilk p < .05) due to one control participant reaching the maximum score at baseline, indicating a ceiling effect. Descriptive inspection nevertheless revealed the same directional pattern as the GGT‑Reha results across all four SLS variants, supporting the overall findings.

### 3.3 Well‑being and physical activity enjoyment

Table 3 displays the pre‑ and post‑test values for well‑being. The analyses revealed neither a significant main effect of time nor a group‑by‑time interaction (F(1, 8) = 0.59, p = .46, ηp² = .07). Table 4 summarizes the results for enjoyment of physical activity. No significant changes were observed over time, and no group‑by‑time interaction was found (F(1, 8) = 0.08, p = .79, ηp² = .01). Descriptive inspection indicated a slight increase in both psychological variables in the intervention group, whereas the control group remained largely unchanged.

**Table 3:** Well-being (M ± SD) before and after the intervention (*n* = 10).

Group	Pre-test	Post-test	*F*	*p*	*ηp2*
Intervention	12.60 ± 6.43	16.00 ± 7.31	0.789	0.425	0.165
Control	13.60 ± 5.41	13.60 ± 1.82	0.000	1.000	0.000

**Table 4:** Physical activity enjoyment (M ± SD) before and after the intervention (*n* = 10).

Group	Pre-test	Post-test	*F*	*p*	*ηp2*
Intervention	64.00 ± 10.42	66.60 ± 13.80	0.543	0.502	0.119
Control	59.00 ± 11.85	55.00 ± 11.75	1.260	0.324	0.240

## 4 Discussion

The present study aimed to examine whether a six‑week balance‑training program could improve static and dynamic balance performance, well‑being, and enjoyment of physical activity among older inmates in an open German prison. In light of demographic changes and the rising proportion of older inmates, developing and evaluating age‑appropriate physical activity interventions within correctional settings is of increasing importance. To the best of our knowledge, this is the first study to implement and evaluate an explicit balance‑training program among older prisoners. It therefore provides new insights into the feasibility and potential benefits of targeted exercise interventions in this unique and understudied population.

Older inmates significantly improved their static balance on both stable and unstable surfaces after the intervention. These improvements of approximately 26% and 33% represent a considerable functional gain within a short period. The results align with earlier findings in community‑dwelling older adults and elderly individuals with a history of falls who also showed enhanced postural control after targeted balance training [29]. This improvement is particularly relevant, given the sedentary prison environment, where sedentary routines and limited spatial freedom often lead to muscular atrophy and loss of coordination. By maintaining or improving postural stability, fall risk among older inmates may be reduced and independence in daily life preserved.

Dynamic balance, in contrast, did not change significantly across groups or measurement points. This result seems plausible because dynamic balance requires more complex motor control, including coordination, reaction speed, and lower‑limb strength. Short‑term interventions rarely produce adaptations in these domains as quickly as in static stability. Studies with longer durations, such as eight‑week [15] or four‑month programs [13], have shown more pronounced effects on dynamic balance and psychological outcomes. Extending training durations to several months may therefore help achieve broader and more sustainable adaptations.

No significant changes were found in well‑being or enjoyment of physical activity following the intervention. However, descriptive data indicated a slight increase in both outcomes within the intervention group, whereas values in the control group remained stable or declined modestly. Although these effects did not reach statistical significance, the direction of change suggests that participation in structured physical activity may support psychological well‑being even in the restrictive prison environment. The short duration of the intervention, the small sample size, and the psychosocial stressors inherent to incarceration likely constrained effects. Given the small sample size (n = 5 per group), the study was underpowered to detect small-to-moderate effects, increasing the risk of a Type II error [30]. Therefore, non-significant findings should be interpreted as inconclusive rather than as evidence of no effect.

This study shows that a short, resource‑efficient balance‑training program can be feasibly implemented in correctional institutions and was associated with measurable functional improvements. The intervention required minimal equipment, achieved good acceptance among participants, and integrated easily into the daily routine of an open prison. These findings appear promising given the accelerated aging and increased fall risk among older inmates. Integrating such health‑oriented exercise programs may help enhance quality of life and could contribute to reducing healthcare costs within correctional systems.

The specific prison context created the main limitations of this study. The most critical constraint was the small sample size, which resulted from the limited number of interested older inmates in the institution. Of about sixty older inmates in the facility, eight volunteered to participate in the balance‑training program, corresponding to a participation rate of approximately 13%. Including the control group, a total of fourteen inmates took part, representing about 23% of the eligible prison population. For ethical reasons, no additional participants could be required to join the training. Although the small sample size clearly limits statistical power, this challenge is not uncommon in applied research contexts with inherently small populations. Similar to elite‑level sports research, where participant numbers are naturally restricted, studies in prison settings face comparable structural constraints. Following the recommendations of Hecksteden et al. [31], the study emphasized reliable measurement procedures and transparent reporting to ensure robust findings despite limited sample sizes. Nevertheless, this sample size exceeded those of previous physical‑activity interventions in prisons, and the inclusion of a control group strengthened the study design compared with many earlier investigations (e.g., [32]).

The study also faced measurement limitations because it relied solely on field‑based tests. Although the GGT‑Reha provides a reliable and valid assessment of balance performance under practical conditions, it does not capture detailed biomechanical parameters such as postural sway or neuromuscular activation patterns that could be analyzed in laboratory settings. The use of the GGT‑Reha rather than the standard GGT version was appropriate for the present sample because the latter would have been too demanding. The GGT‑Reha was specifically developed to assess motor balance in adults with movement disorders and has therefore been widely used in rehabilitation and health‑promotion contexts.

Because all other variables met the assumptions for parametric testing, the exclusion of the SLS results ensured methodological consistency and prevented a potential distortion of variance analyses due to violated test assumptions. Although excluded from the inferential analyses, the descriptive SLS results mirrored the GGT‑Reha outcomes across all four variants, reinforcing the overall pattern of findings. The tasks of the SLS and GGT‑Reha partly overlap, as both include single‑leg stances under different conditions (e.g., eyes open and closed). Participants might also have benefited from learning effects, as repeated testing can increase familiarity with the tasks rather than reflect genuine performance changes.

Although six weeks sufficed to observe significant improvements in static balance, longer training periods are likely to yield stronger effects on dynamic balance and psychological variables. Future studies should therefore extend intervention lengths to at least three to twelve months and include larger sample sizes to enhance statistical power and generalizability. Because this study was conducted in a single open German prison and included only older male inmates, the generalizability of the findings is limited. Future research should, therefore, include older female inmates and facilities with different security levels to assess the broader applicability of the results across various correctional settings. In addition, examining other types of physical activity interventions, such as yoga or strength training, could provide further insight into the broader role and benefits of exercise for older incarcerated populations.

Although the intervention was associated with measurable functional improvements, it remains unclear to what extent these effects can be retained over time without continued training. Because regular practice is essential to maintain balance performance and prevent renewed functional decline, this issue was explicitly addressed during the project. The prison sports officer and the participating inmates were instructed in suitable exercise routines and progression options to continue training independently after the study. Recommendations for the acquisition of basic equipment (e.g., balance pads) were also provided to support long‑term implementation. Future follow‑up assessments could help evaluate the persistence of these effects and the feasibility of ongoing balance training within correctional settings.

Based on these findings and limitations, several next steps emerge for future research and practice:

• Longer and progressive interventions: Extend training durations to 12-24 weeks and integrate progressive elements such as dual‑task or perturbation exercises to better target dynamic balance and fall risk [33].
• Follow‑up assessments: Conduct follow‑up testing after 3-6 months to evaluate the maintenance of balance improvements and the feasibility of continued independent practice [21].
• Expanded outcome measures: Include functional and translational indicators such as The Timed Up & Go Test and gait speed where feasible [34].
• Broader populations and settings: Replicate the intervention in facilities with different security levels and among (older) female inmates to enhance generalizability.
• Implementation and scalability: Evaluate training requirements, equipment constraints, cost, and staff workload to inform integration into correctional health programs.

## 5 Conclusion

The six‑week balance‑training program was associated with significant improvements in static balance among older inmates, while dynamic balance and psychological well‑being remained stable. These functional gains indicate that structured physical activity can effectively counteract age‑related decline even within the constraints of prison life. Implementing longer and more comprehensive training programs will likely strengthen these effects and help maintain mobility, autonomy, and quality of life in aging prison populations.

## Ethics statements

No animal studies are presented in this manuscript.

No potentially identifiable images or data are presented in this study.

The studies involving humans were approved by the Ethics Committee of Bielefeld University. The studies were conducted in accordance with the local legislation and institutional requirements. The participants provided their written informed consent to participate in this study.

## Data availability statement

The participants of this study did not provide written consent for their data to be shared publicly; therefore, due to the sensitive nature of the research, supporting data are not available.

## Declaration of the use of generative AI and AI-assisted technologies in scientific writing and in figures, images and artwork

The authors declare that no generative AI or AI‑assisted tools were used to create or modify any figures, images, or artwork. Generative AI tools were used only to assist with language editing, and the authors take full responsibility for the content and integrity of the manuscript.

## Funding

This research did not receive any specific grant from funding agencies in the public, commercial, or not-for-profit sectors.

## CRediT authorship contribution statement

**Milan Dransmann:** Writing – review & editing, Writing – original draft, Project administration, Methodology, Investigation, Formal analysis, Data curation, Conceptualization. **Martin Koddebusch:** Writing – review & editing. **Bernd Gröben:** Writing – review & editing. **Pamela Wicker:** Writing – review & editing.

## Declaration of competing interest

The authors declare that they have no known competing financial interests or personal relationships that could have appeared to influence the work reported in this paper.
